# Comparison of growth factor levels in injectable platelet-rich fibrin obtained from healthy individuals and patients with chronic periodontitis: a pilot study

**DOI:** 10.1186/s12903-024-04301-x

**Published:** 2024-05-03

**Authors:** Bilge Karcı, Hasan Basri Savas

**Affiliations:** 1 Faculty of Dentistry, Department of Periodontology, Alanya Alaaddin Keykubat University, Alanya, Antalya, Turkey; 2 Faculty of Medicine, Department of Biochemistry, Mardin Artuklu University, Mardin, Turkey

**Keywords:** Injectable platelet rich fibrin, Growth factors, White blood cells, Platelets, Chronic periodontitis

## Abstract

**Background:**

This study aimed to assess and compare the concentrations of growth factors, white blood cells (WBCs), and platelets in injectable platelet-rich fibrin (i-PRF) derived from people with healthy periodontal conditions and those with chronic periodontitis.

**Methods:**

Venous blood samples were obtained from 30 patients diagnosed with chronic periodontitis (test group) and 30 participants with healthy periodontal conditions (control group). The i-PRF was then acquired from centrifuged blood. The growth factors (VEGF, IGF-1, TGF-β1, PDGF-BB and EGF) released from the i-PRF samples were compared between groups with ELISA testing. The amounts of WBCs and platelets were also compared.

**Results:**

No significant differences in the concentrations of growth factors were found between the groups (the mean values for the control and test groups were, respectively: IGF: 38.82, 42.46; PDGF: 414.25, 466.28; VEGF: 375.69, 412.18; TGF-β1: 21.50, 26.21; EGF: 138.62, 154.82). The test group exhibited a significantly higher WBC count than the control group (8.80 vs. 6.60, respectively). However, the platelet count did not show a statistically significant difference between the groups (control group 242.0 vs. test group 262.50). No significant correlation was observed between WBC count and growth factor level in either group.

**Conclusions:**

The growth factor levels in i-PRFs did not exhibit significant difference between the two groups. This suggests that the levels of these growth factors may be unaffected by the periodontal disease.

## Background

Periodontitis is a condition characterized by the gradual deterioration of the periodontal ligament and alveolar bone. This is caused by inflammation resulting from the presence of harmful microorganisms around the tooth and the body’s immune response to them [1].

During the typical wound-healing process, clusters of cells, particularly platelets, migrate towards the affected region. Platelet cells initiate and establish a durable blood clot in the wound area. After the clot forms, the growth factors contained in the granules within the platelets are released into the surrounding area to promote the healing process [2]. The presence of significant quantities of platelet-derived growth factor (PDGF) and transforming growth factor-beta (TGF-β) in platelets’ α granules, and their subsequent release into the surrounding environment during the initial stages of wound healing, present compelling evidence for the crucial role that these cells play in the wound-healing process [3].

Platelet-rich fibrin (PRF), a type of second-generation platelet concentrate, is obtained by trapping platelets, abundant leukocytes, and cytokines within a fibrin network. Some of these platelet concentrations include PDGF, insulin-like growth factor-1 (IGF-1), TGF-β, fibroblast growth factor, and vascular endothelial growth factor (VEGF). PRF enhances the healing processes of both soft and hard tissues by elevating the levels of growth factors, such as epidermal growth factor (EGF). Because PRF is derived from the patient’s body, no chance exists of disease transmission or immune response due to its use [4–8].

In recent years, various platelet concentrates have been created and named for the method used, each involving different centrifugation processes. Joseph Choukroun created injectable platelet-rich fibrin (i-PRF) [9]. Unlike other PRF types, i-PRF is a fluid, injectable form. It comprises hematopoietic stem cells and endothelial cells well as leukocytes and thrombocytes. Therefore, it is regarded as not only a platelet concentrate but also a “blood concentrate” [10].

Injecting i-PRF into soft tissue promotes vascularization. It can also be used by blending it with graft materials. The solid and inflexible structure that develops (after a waiting period for polymerization) is referred to as “sticky bone” and is convenient to use in rebuilding techniques [11]. i-PRF is commonly used for periodontal regeneration, root covering, standard scaling and root planing, increasing gingival thickness, and implant surgery [12]. Additionally, PRF has applications in sinus augmentation and various oral surgeries [13, 14].

Research has demonstrated that patient-related factors affect both the amount and the characteristics of PRF. However, little research has assessed the patient-specific variables influencing the quantity of growth factors derived from PRF production [15].

Previous studies have uncovered several potential connections between periodontitis and systemic diseases. The evidence regarding systemic inflammatory responses suggests that they might either stimulate platelets to release growth factors or suppress the healing response by reducing growth factors levels, possibly in response to inflammation [16].

No study has yet investigated the potential impact of the inflammatory condition or the platelet count of patients with chronic periodontitis on the levels of growth factors present and released by i-PRF.

Therefore, the primary objective of this study was to evaluate and compare the concentrations of growth factors in i-PRF samples obtained from healthy individuals and patients diagnosed with chronic periodontitis. Additionally, it aimed to examine the correlation between the growth factor levels in i-PRF and patients’ WBCs and platelet counts.

## Methods

### Participants

This cross-sectional, observational study conducted at Alanya Alaaddin Keykubat University Faculty of Dentistry Department of Periodontology and Biochemistry. The study began after approval was granted by the Akdeniz University Faculty of Medicine Clinical Research Ethics Committee (dated 05.12.2018, protocol# 859). The participants were selected from individuals who applied to the periodontology clinic due to periodontal problems or for routine cleanings from September 2019 to January 2020. The study’s objectives and extent were thoroughly explained, along with comprehensive information about the potential complications of participating. Written informed consent was acquired from every participant. They were then categorized into two groups based on their periodontal health status [17].

Control group: Individuals without periodontal disease.

Test group: Patients diagnosed with stage III and stage IV, grade B generalized severe chronic periodontitis.

Generalized severe chronic periodontitis was defined as individuals who experied bleeding from over 30% of their teeth on probing, with probing depth equal to or greater than 7 mm, clinical attachment loss equal to or greater than 5 mm, and radiographic bone loss of above 30%. The control group consisted of oeriodontally healthy individuals.

The inclusion criteria were:

1. Aged 30 to 65
2. Controlled or no systemic diseases
3. Nonsmoker.

The exclusion criteria were:

1. Uncontrolled systemic diseases
2. Body mass index ≥ 40 kg/m^2^
3. Pregnant
4. Immunosuppressant drug use
5. Smokers and alcohol user
6. Receiving chemotherapy, radiotherapy or corticosteroid, anticoagulant, antiplatelet, or nonsteroidal anti-inflammatory drugs
7. Drug user
8. Received antibiotics treatment in the last 6 months
9. Received surgical or nonsurgical periodontal therapy for periodontitis within 2 years [18].

### Preparation for PRF

After the demographic data were collected and a periodontal examination was conducted, the patients were provided with follow-up numbers, and the i-PRF collection tubes were labeled with tracking numbers. Two blood samples were collected from the antecubital veins of each participant. One tube was used to prepare the i-PRF, and the other was designated for whole blood, containing WBCs and platelets.

A total of 12 mL of whole blood was collected. Of this, 3 mL were used in the complete blood count analysis, and 9 mL were used for i-PRF centrifugation. The time needed to fill the sample tubes with blood was measured with a timer during collection. The blood extraction procedure was completed in a maximum of 25 s. The blood samples were promptly deposited in centrifuge equipment (within 60 s).

The blood was transferred to plastic i-PRF tubes containing no anticoagulant. The sample was subjected to fixed-angle centrifugation in a tabletop centrifuge (ElektroMag M 415P, Istanbul, TR) at 700 rpm (60 g) for 3 min. After centrifugation, the blood formed two distinct layers. The lower stratum comprised red blood cells, and the top stratum comprised plasma, platelets, and coagulation factors. The separated plasma and platelets were pale yellow. A Pasteur pipette was used to meticulously extract the uppermost layer. This aspirate is a partially active injectable type of platelet-rich fibrin. The i-PRF samples were transferred to Eppendorf tubes. The sample was kept at -80 °C until the day of analysis.

### Evaluation of the complete blood count

Blood samples were collected in tubes containing ethylenediaminetetraacetic acid (EDTA). Automated hematology analyzers were used to measure the complete blood count, which quantifies the various blood cells within a specified volume of blood (Sysmex xn-1000, Japan). We analyzed WBCs and platelets.

### Molecular analysis of biological substances

The levels of TGF-β, human IGF-1, human PDGF-BB, human EGF, and human VEGF-A in the samples were analyzed in duplicate using the ELISA method (Elabscience, Texas, USA [19, 20]. After the completion of the reactions, absorbance measurements were conducted with a microplate reader instrument (Biotek Synergy H1, VT, USA). A concentration vs. optical density plot was generated with the seven-level standard for each parameter. The concentrations of TGF-β, human IGF-1, human PDGF-BB, human EGF, and human VEGF-A in the samples were determined with this graph. The kit specifications for each parameter, including sensitivity, evaluation range, specificity, and repeatability, were: TGF-β: 0.1 ng/mL; 1.16–10 ng/mL, 100% specificity, and coefficient of variation (CV) less than 10%; human IGF-1: 0.94 ng/mL, 1.56–100 ng/mL, 100% specificity, and CV less than 10%; human PDGF-BB: 18.75 pg/mL; 31.2–2000 pg/mL, 100% specificity, and CV less than 10%; human EGF: 2.35 pg/mL, 3.91–250 pg/mL, 100% specificity, and CV less than 10%; and human VEGF-A: 18.75 pg/mL; 31.2–2000 pg/mL, 100% specificity, and CV less than 10%.

### Data analysis

The statistical analyses were conducted with SPSS 19.0 (IBM Inc., IL, USA). The normality of the distribution was assessed with the Kolmogorov–Smirnov and Shapiro–Wilk tests. A significance level of 0.05 was employed. Independent t-tests were used to compare the groups because the data adhered to a normal distribution.

We performed Pearson’s correlation analysis to explore the associations between the growth factor levels and the WBC and platelet counts. The correlation were compared between the test and control groups.

According to the power analysis, with a sample size of 30 in each group, a two-tailed t-test, a significance level of 0.05, and an effect size of 0.75, we could expect around 80% power to detect the effect. This indicates a high probability that the study would detect a difference between the means of the two groups if such a difference existed.

## Results

### Demographic findings

The study involved a cohort of 60 participants, comprising 23 men and 37 women. The mean age was 48.93 ± 3.66 years. The study found no statistically significant difference in age between the groups. The test group’s average age was 52.5 ± 3.46 years, and the control group’s average age was of 45.36 ± 3.73 years (*p* = 0.332). The gender distribution between the two groups showed no notable disparity. The demographic information of the participants is presented in Table 1.

**Table 1:** Participants’ demographic characteristics

		Control	Test		
		***N*(**%**)**			*p*-value
Sex	Male	12 (40.0)	11 (36.7)		0.560
	Female	18 (60.0)	19 (63.3)		
		**Mean ± standard deviation**			
Age		45.36 ± 3.73	52.50 ± 3.46		0.332

### Growth factor levels

**Fig. 1:**
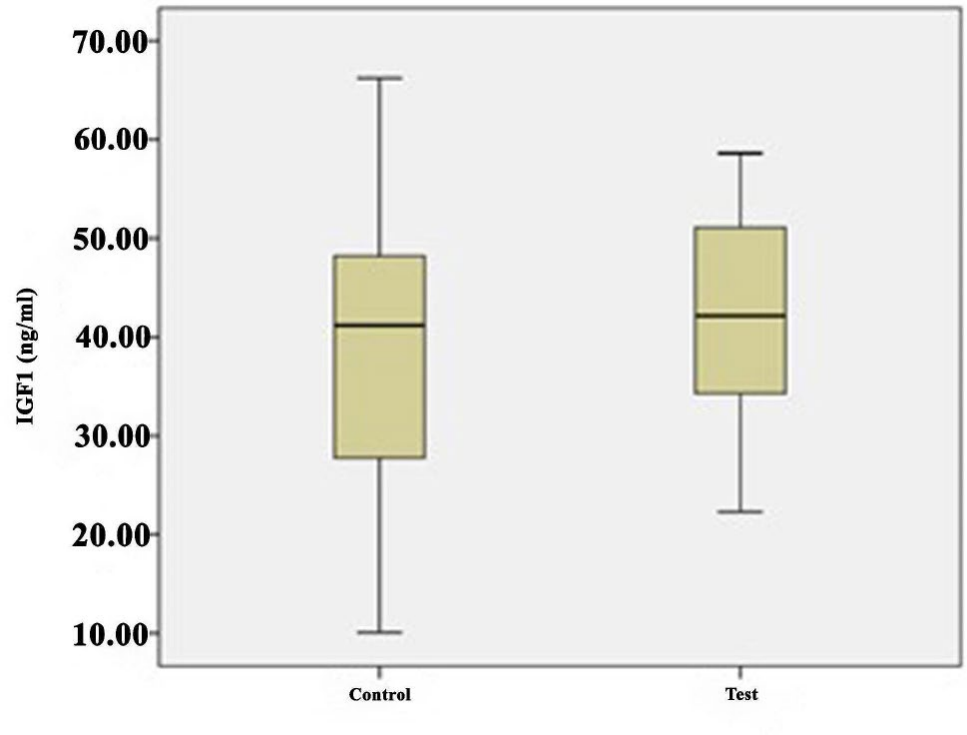
Comparison of insulin-like growth factor (IGF)1 levels between the groups

**Fig. 2:**
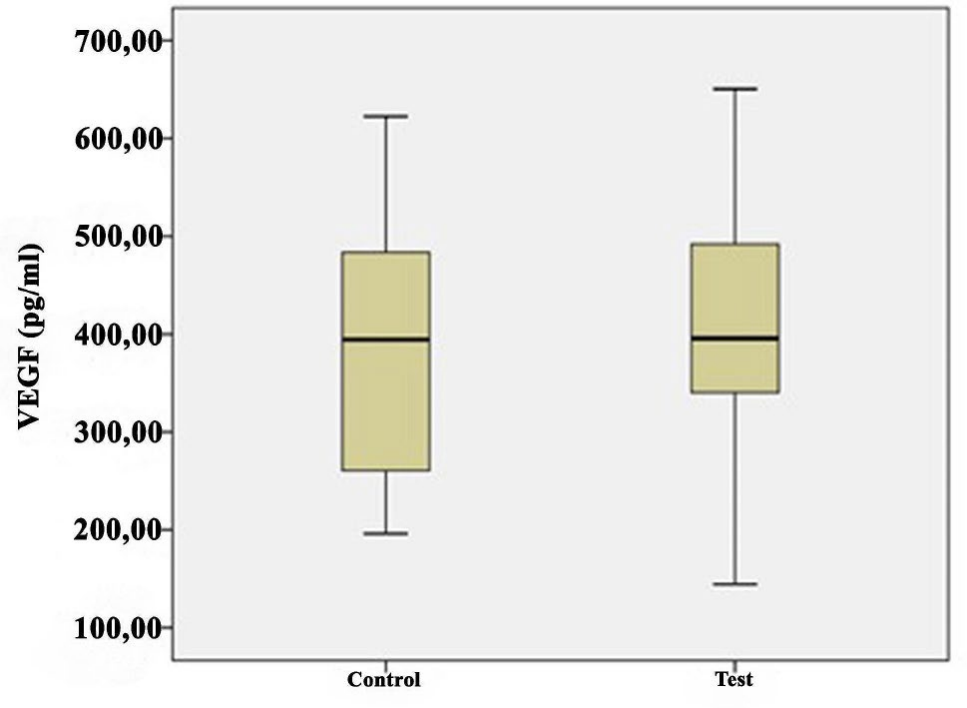
Comparison of vascular endothelial growth factor (VEGF) levels between the groups

**Fig. 3:**
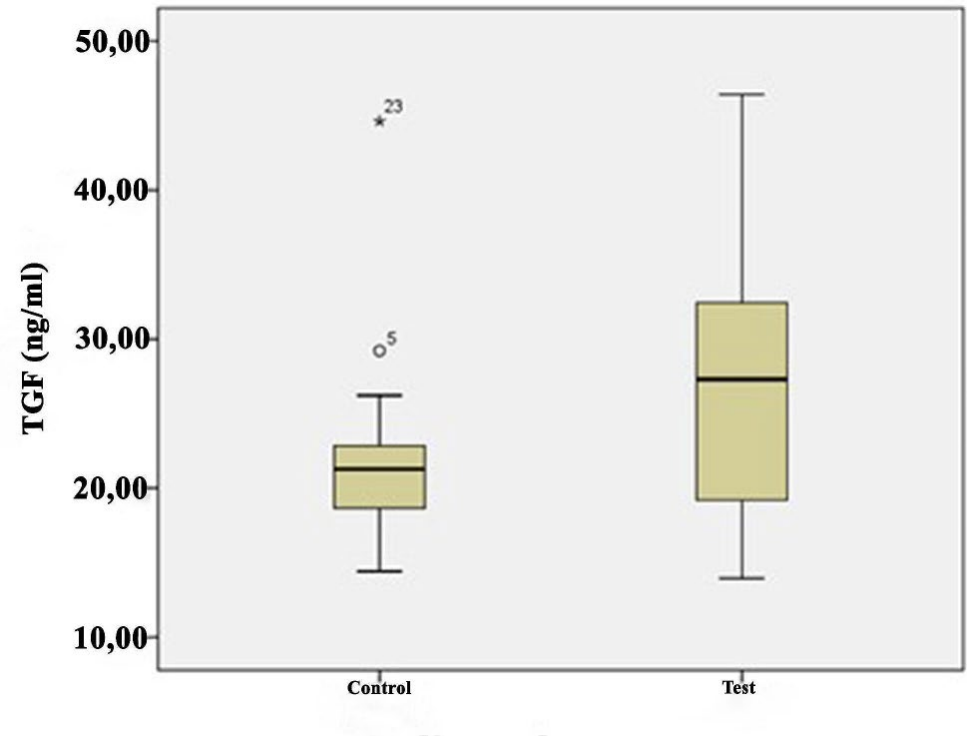
Comparison of transforming growth factor (TGF)-β1 levels between the groups

**Fig. 4:**
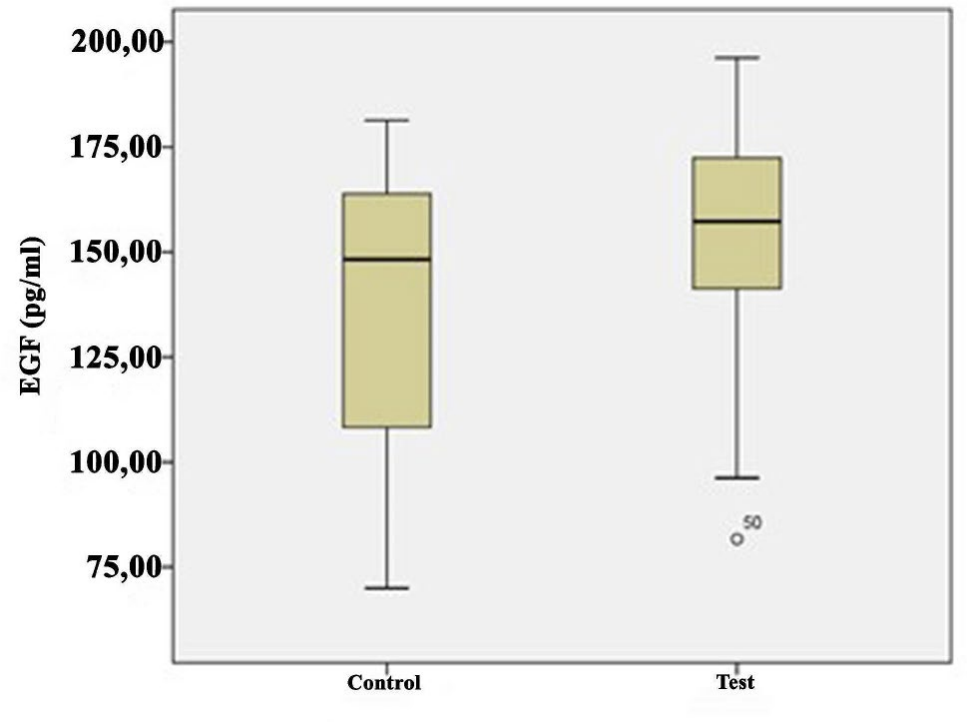
Comparison of endothelial growth factor (EGF) levels between the groups

**Fig. 5:**
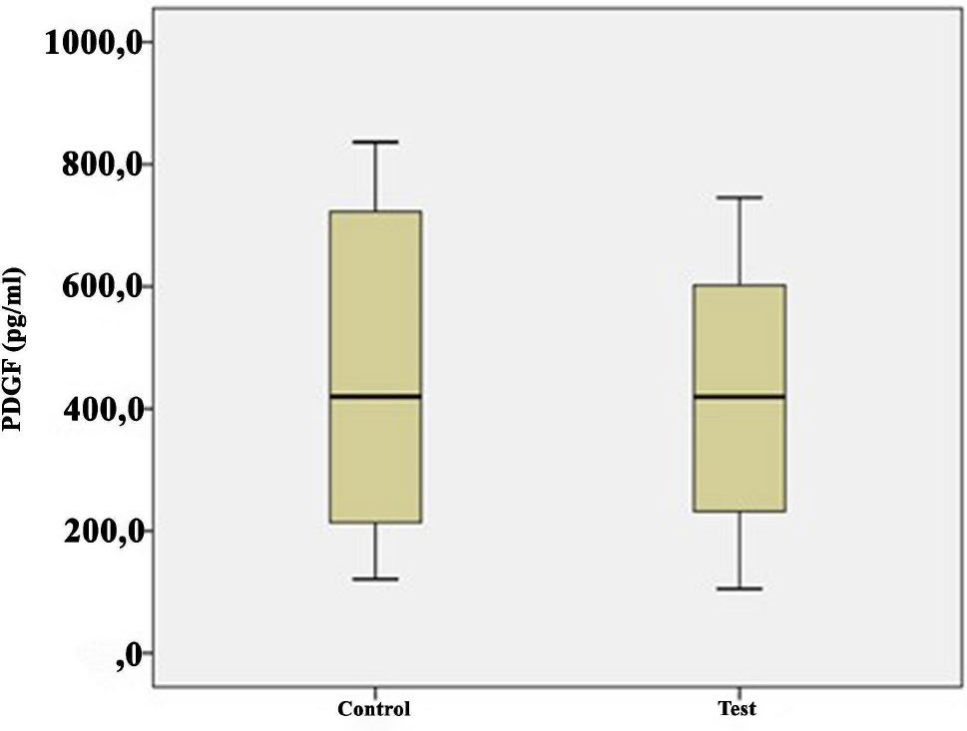
Comparison of platelet-derived growth factor (PDGF)-BB levels between the groups

The growth factor levels in the i-PRF samples are shown in Table 2 and illustrated in Figs. 1, 2, 3, 4 and 5. The levels of VEGF (*p* = 0.084), IGF-1 (*p* = 0.268), TGF-β1 (*p* = 0.121), PDGF-BB (*p* = 0.388), and EGF (*p* = 0.069) in the i-PRF of the test and control groups showed no significant differences. The concentrations of VEGF, IGF-1, TGF-β1, PDGF-BB, and EGF were elevated in the test group compared to the control group. However, this disparity groups did not reach statistical significance (*p* > 0.05).

**Table 2:** Distribution of growth factor levels in i-PRF

		*n*	Mean	Standard deviation	Min	Max	Median	Q1	Q3	t	*P*
IGF1 (ng/mL)	Control	30	38.82	14.99	10.08	66.22	41.18	27.77	60.43	−1.117	0.268
	Test	30	42.46	9.69	22.30	58.60	42.15	34.30	57.27		
PDGF-BB (pg/mL)	Control	30	414.25	107.19	120.74	745.22	419.65	213.60	725.11	0.869	0.388
	Test	30	466.28	102	105.21	836,41	419.24	231.46	822.45		
VEGF (pg/mL)	Control	30	375.69	76.10	196.25	622.52	369.36	250.54	620.66	1.737	0.084
	Test	30	412.18	68.71	144.51	650.50	396.66	294.06	748.35		
TGF-β1 (ng/mL)	Control	30	21.50	8.11	14.41	44.62	20.40	15.45	30.16	1.478	0.121
	Test	30	26.21	7.67	13.94	46.42	25.82	22.95	42.15		
EGF (pg/mL)	Control	30	138.62	22.39	69.36	181.23	155.00	1008.43	177.12	1.854	0.069
	Test	30	154.82	40.97	81.66	196.32	157.59	145.26	192.75		

Q1, 25th percentile; Q3, 75th percentile; Independent-samples t-test

### Comparative analysis of white blood cells (WBC) and platelets

The test group had a markedly elevated WBC count compared to the control group (*p* = 0.025). However, the data obtained by analyzing whole-blood samples, which included the platelet count, did not show any statistically significant differences between the groups (*p* = 0.387) (Table 3). Furthermore, no correlation was observed between WBCs and the growth factors examined in either the test or control groups (*p* > 0.05).

**Table 3:** WBC and platelet values

	Median (Q1–Q3)	Test	*p*-value
	Control		
WBC (cells/mm^3^)	6.60 (5.25–7.55)	8.80 (7.38–9.78)	0.025^*^
Thrombocyte (cells/mm^3^)	242.00 (216–287.75)	262.50 (230.75–298)	0.387

Q1, 25th percentile; Q3, 75th percentile

^*^The test group had a significantly higher WBC value than the control group (*p* < 0.05)

## Discussion

The levels of growth factors (IGF-1, EGF, VEGF, PDGF-BB, and TGF-1) released from i-PRF samples varied significantly among individuals. However, no significant differences were observed between the control and test groups. This suggests that the levels were not influenced by the participants’ periodontal disease status. These findings support the clinical usage of i-PRF to deliver autologous growth factors.

No correlation was observed between participants’ WBC counts and the growth factor levels across the groups, although the data indicated higher WBC levels in the test group. The elevated presence of leukocytes in the test group indicated the expected inflammatory condition. The test group consisted of patients who had been diagnosed with generalized severe chronic periodontitis [21]. No significant differences between the groups were identified from the whole-blood analysis. These results were expected because all patients either had managed systemic disorders or were systemically healthy.

Multiple PRF preparation protocols have been developed. The PRF centrifugation procedure used in this study effectively traps leukocytes and platelets within the fibrin clot [22]. However, TGF-β1, EGF, VEGF, IGF-1, and PDGF-BB primarily originate from platelets, rather than leukocytes [23]. Therefore, we expected no correlation between WBC count and the levels of growth factors derived from PRF. No information is currently available on the influence of complete blood count and the presence of chronic periodontitis on the quantity or release of growth factors from i-PRF.

Maurao et al. [11] achieved a distinct platelet i-PRF preparation using horizontal centrifugation in a B-40 centrifuge (RDE, Brazil) for 2 min at 3300 rpm. The tubes used in this process contained 9 mL of venous blood and were not treated with any anticoagulants. Miron et al. [10] obtained i-PRF by centrifuging 10 mL of whole blood without anticoagulant at 700 rpm for 3 min at room temperature with a Duo Centrifuge, a device specifically designed for obtaining PRF and manufactured in Nice, France. i-PRF is widely accepted to contain not only leukocytes and thrombocytes, but also mesenchymal stem cells and endothelial cells. Therefore, it is considered a blood concentrate rather than solely a platelet concentrate [9].

Chang et al. [18] compared the release of growth factors, WBC count, and thrombocyte level in the PRF membrane and exudate of both periodontally healthy people (control group) and patients with chronic periodontitis (test group). No significant differences were reported in terms of growth factor quantity between the groups. The test group exhibited a markedly elevated WBC count, but no correlation was found among the WBC count, platelet count, and growth factor levels. These findings are consistent with our results.

Kobayashi et al. [24] investigated the growth factors found in the platelet-rich plasma (PRP), PRF, and advanced platelet-rich fibrin (A-PRF) obtained from healthy people. Using ELISA, the amounts of growth factors such as TGF-β, IGF, VEGF, EGF, PDGF-AA, PDGF-BB, and PDGF-AB were measured and released from platelet concentrates over 10 days. The authors hypothesized that the patients’ relationships could affect the growth factor release. The amounts of growth factors released varied significantly. In contrast, we found that the quantity of growth factors in i-PRF was not affected by periodontitis. This difference may reflect the different PRF types produced in these studies.

Miron et al. [10] compared the growth factors found in i-PRF and PRP. They reported that PRP initially produced more growth factors whereas i-PRF released higher levels of PDGF-AA, PDGF-AB, EGF, and IGF over a longer period. We evaluated only one time point. Measuring changes over time or at multiple time points would have been ideal.

In a study comparing i-PRF with PRP in gingival fibroblasts on titanium-implant surfaces, Wang et al. [25] found higher levels of PDGF and TGF-β in the i-PRF group. Furthermore, the i-PRF group had higher levels of cellular migration and collagen-1. We solely examined whether the growth factor levels in i-PRF were affected by periodontitis.

In their investigation of the release of leukocytes, platelets, and growth factors from liquid PRF products, Choukroun et al. [26] found that low-speed centrifugation increased these components in PRF-based matrices. Varela et al. [27] assessed the release of growth factors from i-PRF, the gene expression of type I collagen, its morphological characteristics, and its cellular composition. According to their work, i-PRF, which includes platelets, growth factors, type I collagen, leukocytes, and osteocalcin, may effectively promote the healing of both hard and soft tissues.

The ELISA technique is a highly sensitive biochemical measurement method based on antigen-antibody binding. The limitations of this method may include the researcher’s skill in protocol implementation, the construction of a concentration curve using standards, and the subsequent indirect calculation of sample concentrations based on this curve [28].

The release of growth factors from PRF varied among patients. However, the levels of growth factors derived from the i-PRF did not exhibit a statistically significant difference between the test and control groups. The complete blood count results indicated that the test group exhibited a statistically significant increase in WBC. However, no substantial correlation was observed between WBC counts and growth factors levels in i-PRF.

This study had several limitations.

1. The specific acquisition of i-PRF and the absence of evaluation of other types of PRF.
2. The determination of participants’ systemic disease relied solely on their self-reporting.
3. The sample size was limited.
4. Only one time point assessment was made. Measuring changes over time or collecting data at multiple time points would have been ideal.
5. Evaluation before and after periodontal treatment was not conducted for the periodontitis group.

## Conclusions

The quantity of growth factors in i-PRF was not affected by periodontitis. Within the limitations of this study, these results affirm that i-PRF can be used as a self-derived source of growth factors that remain unaffected by the periodontal condition of systemically healthy individuals. Future studies with larger sample sizes will enable us to understand how the growth factors in i-PRF are affected by periodontitis.

## Data Availability

It can be requested from the corresponding author if deemed necessary.

## References

[1] Ohlrich EJ, Cullinan MP, Seymour GJ (2009). The immunopathogenesis of periodontal disease. Aust Dent J.

[2] Anitua E, Andia I, Ardanza B, Nurden P, Nurden AT (2004). Autologous plateletes as a source of proteins for healing and tissue egeneration. Thromb Heamostasis.

[3] Anitua E, Sánchez M, Orive G, Andía I (2007). The potential impact of the preparation rich in growth factors (PRGF) in different medical fields. Biomaterials.

[4] Choukroun J, Diss A, Simonpieri A (2006). Platelet-rich fibrin (PRF): a second-generation platelet concentrate. Part IV: clinical effects on tissue healing. Oral Surg Oral Med Oral Pathol Oral Radiol Endod.

[5] Choukroun J, Diss A, Simonpieri A, Girard MO, Schoeffler C, Dohan SL (2006). Platelet-rich fibrin (PRF): a second-generation platelet concentrate. Part V: histologic evaluations of PRF effects on bone allograft maturation in sinus lift. Oral Surg Oral Med Oral Pathol Oral Radiol Endod.

[6] Dohan DM, Choukroun J, Diss A, Dohan SL, Dohan AJ, Mouhyi J (2006). Platelet-rich fibrin (PRF): a second-generation platelet concentrate. Part I: technological concepts and evolution. Oral Surg Oral Med Oral Pathol Oral Radiol Endod.

[7] Dohan DM, Choukroun J, Diss A, Dohan SL, Dohan AJ, Mouhyi J (2006). Platelet-rich fibrin (PRF): a second-generation platelet concentrate. Part II: platelet-related biologic features. Oral Surg Oral Med Oral Pathol Oral Radiol Endod.

[8] Dohan DM, Choukroun J, Diss A, Dohan SL, Dohan AJ, Mouhyi J et al. Platelet-rich fibrin (PRF): a second-generation platelet concentrate. Part III: leucocyte activation: a new feature for platelet concentrates? Oral surg oral Med oral pathol oral Radiol Endod. 2006; 101(3):51–5. 10.1016/j.tripleo.2005.07.010. 10.1016/j.tripleo.2005.07.010 16504851

[9] Choukroun J, Advanced PRF (2014). i-PRF: platelet concentrates or blood concentrates. J Periodontal Med Clin Pract.

[10] Miron RJ, Fujioka-Kobayashi M, Hernandez M, Kandalam U, Zhang Y, Ghanaati S (2017). Injectable platelet rich fibrin (i-PRF): opportunities in regenerative dentistry?. Clin Oral Investig.

[11] Mourão CF, de AB, Valiense H, Melo ER, Mourão NBMF, Maia MD-C (2015). Obtention of injectable platelets rich-fibrin (i-PRF) and its polymerization with bone graft: technical note. Rev Col Bras Cir.

[12] Miron RJ, Gruber R, Farshidfar N, Sculean A, Zhang Y (2023). Ten years of injectable platelet-rich fibrin. Periodontol 2000.

[13] Sabri H, Sarkarat F, Mortezagholi B, Aghajani D (2022). Non-surgical management of oro-antral communication using platelet-rich fibrin: a review of the literature. Oral Surg.

[14] Alrmali A, Saleh MHA, Kurdi SMS, Sabri H, Meghil MM, Wang HL (2023). Prevention and management of drug-induced osteonecrosis of the jaws using platelet-rich fibrin: a clinical feasibility study. Clin Exp Dent Res.

[15] Miron RJ, Dham A, Dham U, Zhang Y, Pikos MA, Sculean A (2019). The effect of age, gender, and time between blood draw and start of centrifugation on the size outcomes of platelet-rich fibrin (PRF) membranes. Clin Oral Investig.

[16] Sabharwal A, Gomes-Filho IS, Stellrecht E, Scannapieco FA (2018). Role of periodontal therapy in management of common complex systemic diseases and conditions: an update. Periodontol.

[17] AAP, American Academy of Periodontology Task Force (2015). American Academy of Periodontology task force report on the update to the 1999 classification of periodontal diseases and conditions. J Periodontol.

[18] Chang J, Blanchard SB, Windsor LJ, Gregory RL, Hamada Y (2020). Levels of growth factors from platelet-rich fibrin from chronic periodontitis versus periodontally healthy subjects: a pilot study. Clin Oral Investig.

[19] Ozturk SA, Ceylan C, Serel TA, Doluoglu OG, Soyupek AS, Guzel A (2015). Protective effect of theophylline on renal functions in experimental pneumoperitoneum model. Ren Fail.

[20] Temelli B, Yetkin Ay Z, Savaş HB, Aksoy F, Kumbul Doğuç D, Uskun E et al. Circulation levels of acute phase proteins pentraxin 3 and serum amyloid A in atherosclerosis have correlations with periodontal inflamed surface area. J Appl Oral 2018; Sci 26:e20170322. 10.1590/1678-7757-2017-0322. 10.1590/1678-7757-2017-0322 PMC5933826 29742255

[21] Ehrenfest DMD, Rasmusson L, Albrektsson T (2009). Classification of platelet concentrates: from pure platelet-rich plasma (P-PRP) to leucocyte-and platelet-rich fibrin (L-PRF). Trends Biotechnol.

[22] Del Corso M, Vervelle A, Simonpieri A, Jimbo R, Inchingolo F, Sammartino G, Ehrenfest DMD (2012). Current knowledge and perspectives for the use of platelet-rich plasma (PRP) and platelet-rich fibrin (PRF) in oral and maxillofacial surgery part 1: periodontal and dentoalveolar surgery. Curr Pharm Biotechnol.

[23] Larjava H. Oral wound healing: cell biology and clinical management. 1rd ed. Wiley; 2012. pp. 261–86.

[24] Kobayashi E, Flückiger L, Fujioka-Kobayashi M, Sawada K, Sculean A, Schaller B. at al. Comparative release of growth factors from PRP, PRF, and advanced-PRF. Clin Oral Investig. 2016;20(9):2353–60. 10.1007/s00784-016-1719-1. 10.1007/s00784-016-1719-1 26809431

[25] Wang X, Zhang Y, Choukroun J, Ghanaati S, Miron RJ (2017). Behavior of gingival fibroblasts on titanium implant surfaces in combination with either Injectable-PRF or PRP. Int J Mol Sci.

[26] Choukroun J, Ghanaati S (2018). Reduction of relative centrifugation force within injectable platelet-richfibrin (PRF) concentrates advances patients’ own inflammatory cells, platelets and growth factors: the first introduction to the low speed centrifugation concept. Eur J Trauma Emerg Surg.

[27] Varela HA, Souza JCM, Nascimento RM, Araújo RF, Vasconcelos RC, Cavalcante RS (2019). Injectable platelet rich fibrin: cell content, morphological, and protein characterization. Clin Oral Investig.

[28] Lai S, Wang S, Luo J, Lee LJ, Yang S-T, Madou MJ (2004). Design of a compact disk-like microfluidic platform for enzyme-linked immunosorbent assay. Anal Chem.

